# A Series of Beta-Carboline Derivatives Inhibit the Kinase Activity of PLKs

**DOI:** 10.1371/journal.pone.0046546

**Published:** 2012-10-03

**Authors:** Xiaomin Han, Jing Zhang, Liang Guo, Rihui Cao, Yongzhen Li, Ni Li, Qin Ma, Jialin Wu, Yanchang Wang, Shuyi Si

**Affiliations:** 1 Institute of Medicinal Biotechnology, Peking Union Medical College & Chinese Academy of Medical Sciences, Beijing, People’s Republic of China; 2 Xinjiang Huashidan Pharmaceutical Co., Urumqi, People’s Republic of China; 3 Department of Biomedical Sciences, College of Medicine, Florida State University, Tallahassee, Florida, United States of America; University of Pittsburgh School of Medicine, United States of America

## Abstract

Polo-like kinases play an essential role in the ordered execution of mitotic events and 4 mammalian PLK family members have been identified. Accumulating evidence indicates that PLK1 is an attractive target for anticancer drugs. In this paper, a series of beta-carboline derivatives were synthesized and three compounds, DH281, DH285 and DH287, were identified as potent new PLK inhibitors. We employed various biochemical and cellular approaches to determine the effects of these compounds on the activity of PLK1 and other mitotic kinases and on cell cycle progression. We found that these three compounds could selectively inhibit the kinase activity of purified PLK1, PLK2 and PLK3 *in vitro.* They show strong antitumor activity against a number of cancer cell lines with relatively low micromolar IC_50_s, but are relatively less toxic to non-cancer cells (MRC5). Moreover, these compounds could induce obvious accumulation of HeLa cells in G_2_/M and S phases and trigger apoptosis. Although MRC5 cells show clear S-phase arrest after treatment with these compounds, the G2/M arrest and apoptosis are less insignificant, indicating the distinct sensitivity between normal and cancer cells. We also found that HeLa cells treated with these drugs exhibit monopolar spindles and increased Wee1 protein levels, the characteristics of cells treated with PLK1 inhibitors. Together, these results demonstrate that DH281, DH285 and DH287 beta-carboline compounds are new PLK inhibitors with potential for cancer treatment.

## Introduction

Polo-like kinases (PLKs) are a family of serine-threonine kinases with a kinase domain at the N-terminus followed by one or two C-terminal polo-box domains that are involved in substrate binding [1]. Among the four members of PLKs in mammalian cells, PLK1 is the best characterized and is recognized to be a key component of the cell cycle machinery with important roles in mitotic entry [2], centrosome duplication [3], bipolar mitotic spindle formation, metaphase to anaphase transition, cytokinesis and maintenance of genome stability [4]. PLK1 is highly expressed in proliferating cancer cells, including breast cancer [5], colorectal cancer [6], esophagus and stomach cancer [7], endometrial carcinomas [8], head and neck squamous cell carcinomas [9], non-small cell lung cancer [10], ovarian cancer [11], pancreatic cancer [12] and skin cancer [13] etc. In some types of tumors, overexpression of PLK1 correlates with a poor prognosis. Down-regulation of PLK1 activity has been shown to inhibit cell proliferation of cancer cell lines [14], [15] and tumor xenografts [16]. Moreover, interfering with PLK1 activity by a variety of methods, including antisense oligonucleotides, small interfering RNA and various dominant negative agents, leads to apoptosis in both cell culture and animals [16], [17], [18], [19], [20], [21]. Interestingly, normal cells but not tumor cells can survive from PLK1 depletion [22], thus PLK1 is a promising target for antitumor therapy. Both PlK2 and PLK3 are the members closely relative to PLK1 in the PBD domain. However, the function of PLK2 and PLK3 remains unclear, in cancer cells PLK2 and PLK3 exist as important mediators of stress phenotypes in response to DNA damage or oxidative stress [23]. PLK4 is the member distinct from PLK1 in the PBD domain, but PLK4 is also essential for cell division. The role of PLK4 in centriole duplication is well established and silencing of PLK4 results in disorganized mitotic spindles and apoptosis [24].

Increasing efforts have been made to identify small-molecule PLK inhibitors for preclinical development and clinical trials. A complete list of PLK inhibitors in development has been summarized [25]. All of them can be divided into non-ATP-competitive and ATP-competitive small-molecule inhibitors [26]. BI2356 [27], GSK461364 [28], ON01910 [29], and HMN-214 [30] are the four extensively studied PLK inhibitors that are undergoing phase I or II trials.

We are interested in isolating new small-molecule PLK1 inhibitors. As PLK1 is a conserved protein kinase, we believe its yeast homologue Cdc5 should be sensitive to PLK1 inhibitors as well. Given that temperature sensitive *cdc5* mutants exhibit compromised Cdc5 kinase activity even at the permissive temperature [31], the mutant cells are expected to be more sensitive to PLK inhibitors. Based on this rationale, we have previously identified DH166 (phenylpropyl-1-methyl-7-methoxyl-9-(3-chlrophenyl)-β-carboline), which turns out to be a novel and moderate ATP-competitive PLK1 inhibitor. We further showed that DH166 inhibited the proliferation of several tumor cell lines [32]. The identification of DH166 as a PLK1 inhibitor prompted our further investigation into this class of compounds. We synthesized additional 18 beta-carboline derivatives and examined the growth inhibition of several non-cancer and cancer cell lines as well as their activities against PLK1 and other kinases. Three compounds, DH281, DH285 and DH287 show strong anti-PLK activity and growth inhibition of cancer cells, suggesting that they are new PLK inhibitors.

## Results

### Antitumor Activity of the 18 Beta-carboline Derivatives

**Figure 1:**
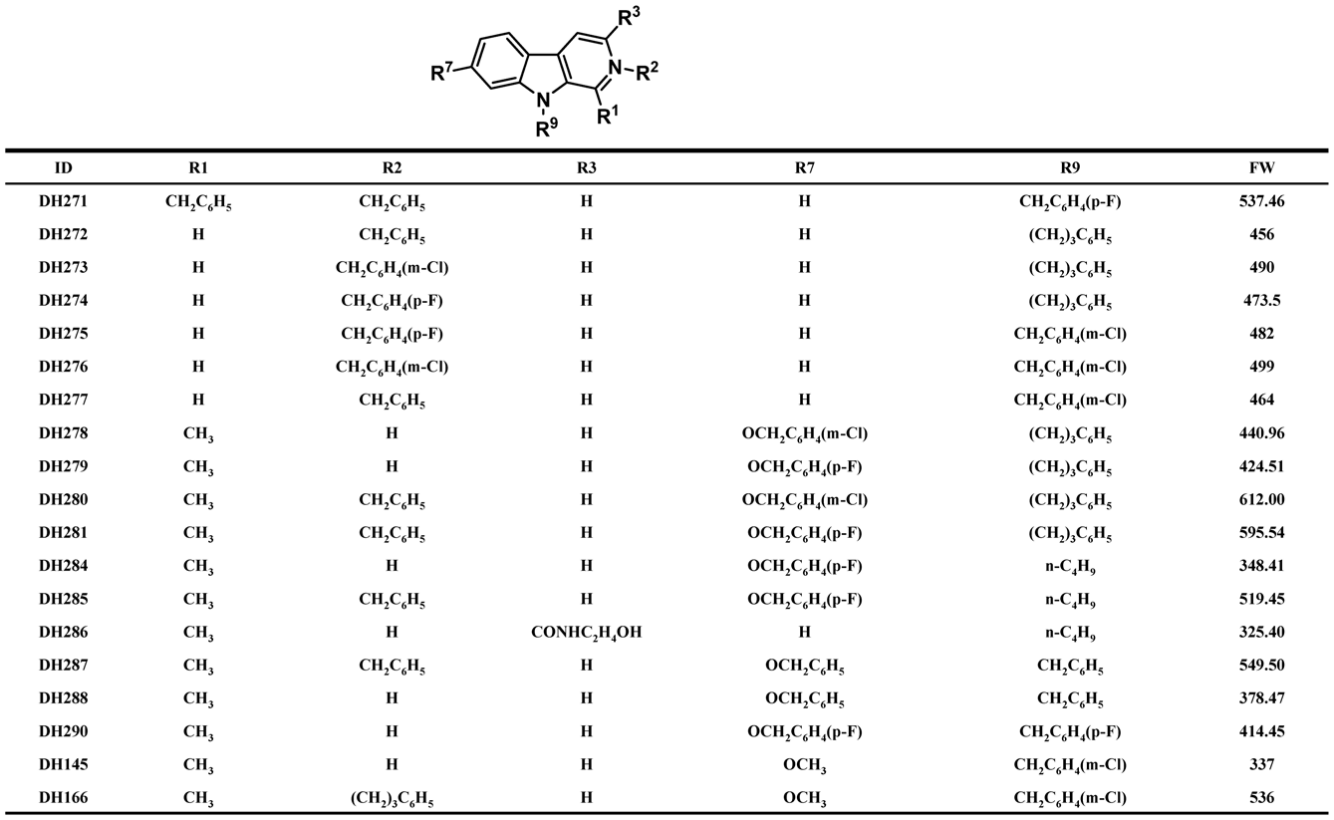
The structures of the beta-carboline derivatives.

We have identified DH166, a beta-carboline derivative, as a PLK1 inhibitor, and this compound shows antitumor activity [32]. In order to find more efficient antitumor small molecules targeting PLK1, we synthesized additional 18 beta-carboline compounds and the structures of these compounds are shown in Figure 1. The growth inhibition of four cancer cell lines (HepG2, MG63, HeLa and PC3) by these compounds was examined. Among these compounds, DH145, DH278, DH279, DH284, DH286, DH288 and DH290 did not show obvious antitumor activity. In contrast, DH280, DH281, DH285 and DH287 exhibited very strong growth inhibition for these cancer cell lines. Other compounds exhibited mild antitumor activity with relatively high IC_50_ values (Table 1). We selected DH281, DH285 and DH287 for further studies.

**Table 1:** The IC_50_s (µM) of the 18 beta-carboline derivatives for cancer and non-cancer cells.

Compound ID	HepG2	MG63	HeLa	PC3	MRC5
DH145	68.45±0.02	73.12±0.31	40.50±0.14	75.39±0.16	ND
DH271	4.01±0.10	5.97±0.06	3.86±0.44	8.38±0.24	ND
DH272	12.47±0.12	12.84±0.28	8.16±0.37	22.07±0.43	ND
DH273	5.08±0.38	4.21±0.45	3.77±0.25	3.86±0.12	ND
DH274	7.37±0.26	6.38±0.19	5.73±0.18	6.07±0.27	ND
DH275	10.42±0.37	7.32±0.22	5.83±0.03	6.36±0.11	ND
DH276	11.22±0.22	7.30±0.06	6.04±0.17	6.65±0.40	ND
DH277	18.76±0.29	20.40±0.16	8.48±0.23	10.83±0.21	ND
DH278	>100	>150	63.14±0.47	80.21±0.31	ND
DH279	>100	>100	>100	>100	ND
DH280	2.59±0.16	1.86±0.33	0.99±0.14	2.34±0.06	ND
DH281	2.24±0.04	1.60±0.15	0.51±0.08	0.94±0.23	4.73±0.51
DH284	59.36±0.46	67.34±0.37	38.29±0.77	27.25±0.89	ND
DH285	11.27±0.40	1.90±0.21	1.27±0.05	2.13±0.07	2.71±0.44
DH286	>150	46.09±0.37	65.81±0.23	72.96±0.53	ND
DH287	10.66±0.31	1.79±0.29	1.82±0.15	2.86±0.19	3.06±0.69
DH288	>100	>100	>100	>100	ND
DH290	51.36±0.20	39.45±0.20	33.30±0.35	31.00±0.38	ND

ND stands for not determined.

### Inhibition of the Kinase Activity of Purified PLK1 by the Beta-carboline Compounds

**Figure 2:**
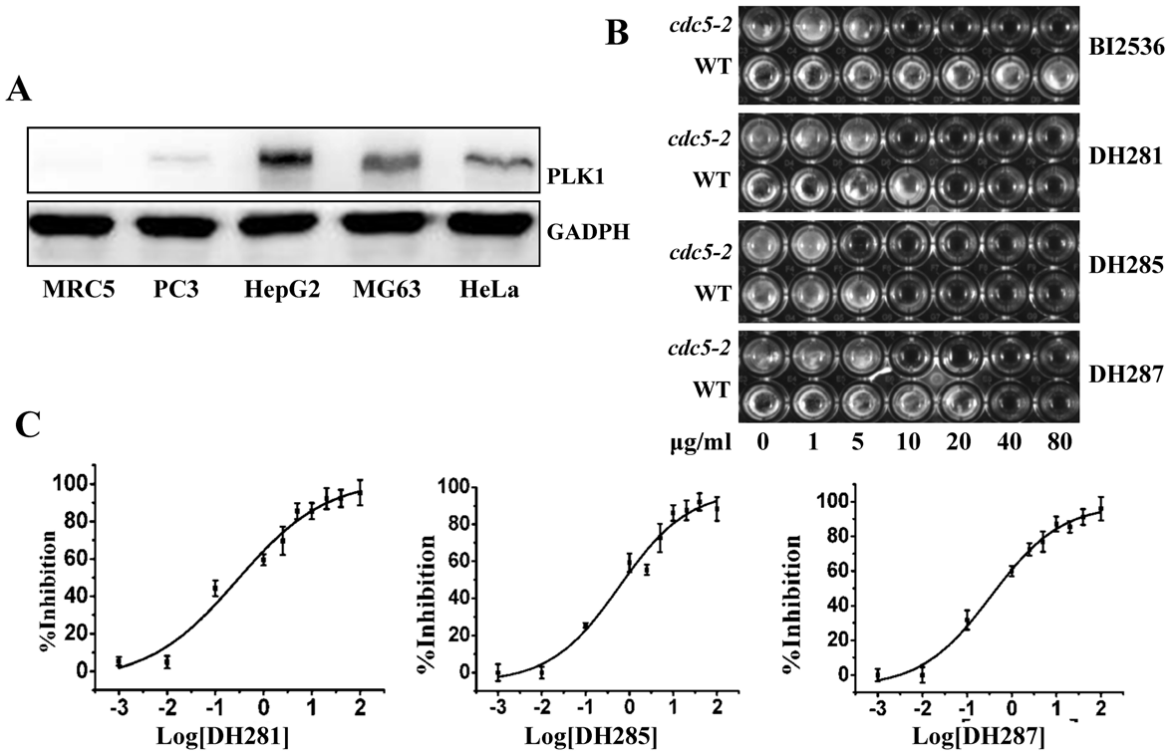
Compounds DH281, DH285 and DH287 are PLK1 inhibitors. **A**. The PLK1 protein levels in cancer and non-cancer cell lines. Protein samples were prepared from the indicated cells lines. After separation with 12% acrylamide gel, the proteins were detected using anti-PLK1 antibody. GADPH was used as a loading control. **B**. *cdc5-2* mutant yeast cells are more sensitive to the β-carboline compound and BI2536 than wild-type cells. Saturated yeast cells (2 µl) were inoculated into 200 µl YPD with indicated concentration of compounds in 96-well plates. The picture was taken after incubation at 25°C for 24 hr. **C**. DH281, DH285 and DH287 inhibit the kinase activity of PLK1. The *in vitro* kinase assay was performed as described in the materials and methods. Inhibitors with different concentrations were used to determine the IC_50_ of these compounds against PLK1. The experiments were repeated for three times and the means and SD are shown.

PLK1 is overexpressed in a wide variety of cancers, and inhibition of this kinase preferentially kills cancer cells over normal cells [22]. Thus, we first compared PLK1 protein levels in the four cancer cell lines (HepG2, MG63, HeLa, and PC3) and a non-cancer cell line MRC5 (Human fetal lung fibroblast). Indeed, MG63, HeLa, and HepG2 cells exhibited an obvious strong PLK1 protein band after Western blotting. PC3 cells also showed a weak but visible PLK1 band, but PLK1 was undetectable in MRC5 cells under this experimental condition (Figure 2A). We then examined the growth inhibition by DH281, DH285 and DH287 using MRC5 cells and the IC_50_s were 4.73, 2.71, and 3.03 µM, respectively. These values are higher than that of MG63, PC3 and HeLa cells. Because PLK1 plays a more important role in the division of cancer cells, we speculate beta-carboline compounds may preferentially inhibit the growth of cancer cells by targeting PLK1. It should be noted that HepG2 cells are less sensitive to these compounds although this cell line showed very high PLK1 protein level, and other unidentified factors might be responsible for its low sensitivity. As HeLa cell line was more sensitive to these compounds compared to others, this cell line was used in the following studies.

To test if these compounds inhibit the growth of cancer cell lines by targeting PLK1, we first compared the sensitivity of wild-type and *cdc5-2* temperature sensitive yeast mutants to these compounds. The Cdc5 kinase is the functional homologue of PLK1 in budding yeast and the incubation of *cdc5-2* mutants at 37°C impairs the kinase activity and stops cell growth. Because the mutated Cdc5-2 protein is not fully functional even at permissive temperature 25°C [31], *cdc5-2* cells are expected to be more sensitive to Cdc5 kinase inhibitors than wild-type cells. As a reported ATP-competitive PLK1 inhibitor, BI2536 showed a complete growth inhibition of *cdc5-2* mutant cells at 10 µg/ml at 25°C, while the growth of wild-type cells was unaffected (Figure 2B). We also compared the sensitivity of wild-type and *cdc5-2* mutant cells to the selected compounds at different concentrations. As shown in Figure 2B, DH281, DH285 and DH287 exhibited stronger growth inhibition of *cdc5-2* compared to wild-type cells, ranging from 2 to 4 fold differences in the MICs (minimum inhibition concentration). Because Cdc5 is the PLK1 homologue and the compromised Cdc5 kinase activity in *cdc5-2* mutant cells leads to increased sensitivity to DH281, DH285 and DH287, these compounds are likely PLK1 inhibitors.

To further test the possibility that these compounds are PLK1 inhibitors, we examined the kinase activity of purified human PLK1 in their presence using ELISA (Enzyme-linked Immunosorbent Assay). DH281, DH285 and DH287 showed strong inhibition of the PLK1 kinase activity in a dose-dependent manner with IC_50_ values at 0.854, 0.310 and 0.527 µM, respectively (Figure 2C), which are lower than the IC_50_ of DH166 (0.897 µM) reported previously [32]. However, other compounds exhibited weaker or no activity, even though they could inhibit the growth of some cancer cells.

Although DH281, DH285 and DH287 showed strong inhibition of PLK1 kinase activity, they may also inhibit other kinases. To clarify this issue, we further examined the kinase activity of PLK2, PLK3, Aurora A, Aurora B, CDK1/cyclinB (human) and CDK2/cyclinE (human) in the presence of these compounds. The IC_50_s for the three compounds against these kinases are listed in Table 2. It was obvious that human CDK1 (associated with cyclin B or cyclin E) and Aurora kinases (A and B) are not very sensitive to the tested beta-carboline compounds. However, both PLK2 and PLK3 exhibited sensitivity to these compounds and the IC_50_s are comparable with that of PLK1. These results support the conclusion that DH281, DH285 and DH287 are the kinase inhibitors specific for the PLK family.

**Table 2:** The IC_50_s (µM) of DH281, DH285, DH287 and DH166 against relevant kinases.

Compound ID	PLK1	PLK2	PLK3	CDK1/cyclinB(h)	CDK2/cyclinE(h)	Aurora-A	Aurora-B
**DH281**	0.854	3.82	1.11	42.1	>100	–	–
**DH285**	0.310	6.45	1.81	70.2	>100	>50	>50
**DH287**	0.527	4.30	0.607	39.4	>100	22.56	24.68
**DH166**	0.897	9.54	3.39	80.3	>100	–	–

### Virtual Docking between the Beta-carboline Compounds and the Kinase Domain of PLK1

**Figure 3:**
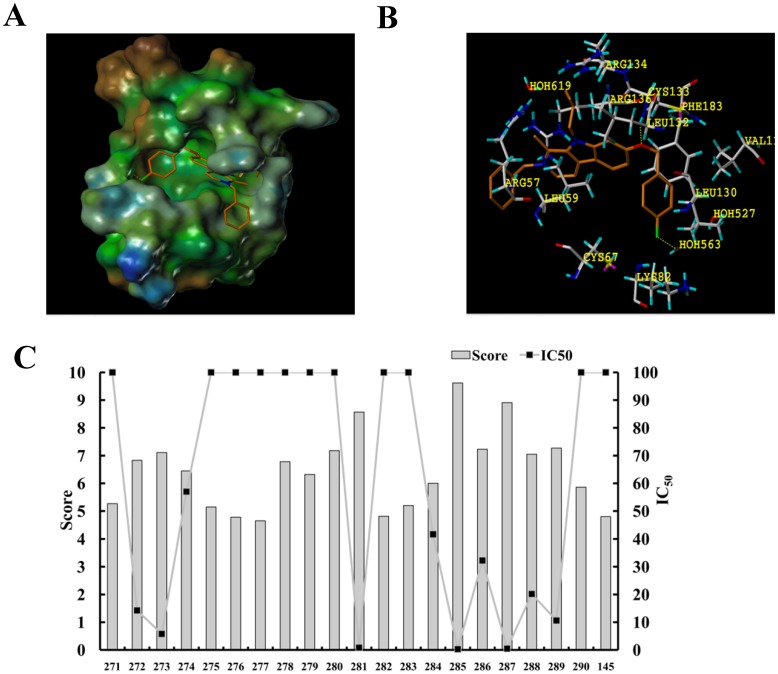
The virtual docking of PLK1 kinase domain with beta-carboline derivatives. A . Overview of the kinase domain of PLK1 with DH285 bound to the active pocket. DH285 is rendered by a stick model (red, oxygen atoms; white, carbon atoms; blue, nitrogen atoms; aqua, chlorine atoms). The molecular surface of the kinase domain is colored with electrostatic potentials and the blue color indicates negatively charged regions. **B**. Specific hydrogen bond (yellow broken lines) formed between DH285 (stick model) and residues (violet ball-and-stick model) in the ATP-binding pocket. The panel A and B were generated by SYBYL7.3 software. **C**. the docking scores of beta-carboline derivatives with the kinase domain of PLK1. The IC_50_ for these compounds against PLK1 is shown.

To determine how the structures of these beta-carboline compounds contribute to their differential inhibitory activity against PLK1 kinase, virtual docking analysis with the kinase domain of PLK1 was performed. The crystal structure of PLK1 solved at 2.1-Å resolution was retrieved from the Protein Data Bank (PDB ID code 2OWB). With score 6 defined as reciprocally matched for protein-ligand complex, compounds DH281, DH285 and DH287 showed highest docking scores (8.57, 9.62, and 8.91) (Figure 3C), while DH166 marked 7.58, which is in agreement with the high anti-PLK1 activity of these compounds. The docking model shows that DH285 binds to the kinase pocket, and the whole beta-carboline molecule is sandwiched into the “C” shape cavity structure between the side chain of Leu59 from the N-terminus and the Arg136 from the C-terminus (Figure 3A). Moreover, two hydrogen bonds are formed; one between the Oxygen atom of p-fluro benzyloxy group, which is located at position 7 of beta-carboline, and the NH group of Leu132. The other is a water-mediated hydrogen bond between F atom and NH from Val129. At the same time, the presence of Phe183 (commonly a leucine or methionine in other kinases) at the binding site further enhances the affinity through π-π stacking with benzene at the p-fluro benzyloxy group (Figure 3B). The virtual docking results indicate that DH281, DH285 and DH287 exhibit strong binding to the kinase domain of PLK1, which could explain their stronger anti-PLK1 activity than the others including DH166. Therefore, these three compounds were used for further detailed analysis.

### DH281, DH285 and DH287 Induce G_2_/M and S Phase Arrest and Apoptosis in HeLa Cells but are Less Effective on the Normal Human Cell Line MRC5

**Figure 4:**
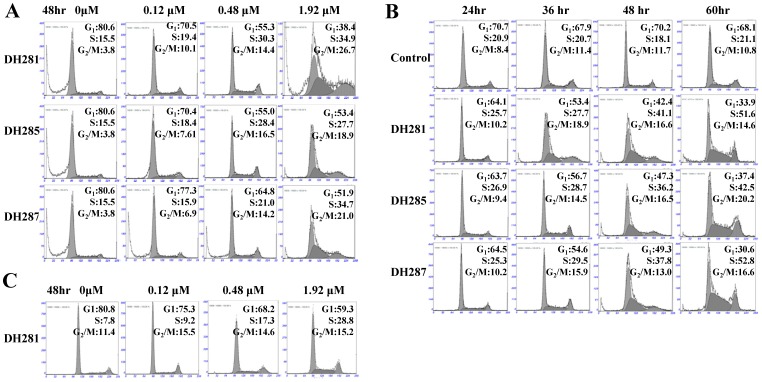
The cell cycle response of HeLa and MRC5 cells to DH281, DH285 and DH287. **A**. HeLa cells treated with 0, 0.12, 0.48, and 1.92 µM compounds for 48 hr were collected for FACS analysis. **B**. HeLa cells were cultured in the presence of DH281, DH285 and DH287 (0.8 µM) for 24, 36, 48, and 60 hr. The cells were collected and stained with PI for FACS analysis. **C**. MRC5 cells treated with 0, 0.12, 0.48, and 1.92 µM DH281 for 48 hr were collected for FACS analysis.

It is well documented that PLK1 is involved in multiple steps in mitosis, such as centrosome maturation and establishment of bipolar spindle [3]. Recent evidence indicates that inhibition of PLK1 also impairs DNA replication and results in slow S-phase progression in cultured cancer cells [33]. Therefore, the down-regulation of PLK1 would change the distribution of cells in different cell cycle stages. To test this possibility, fluorescence-activated cell sorter (FACS) analysis was used to analyze the DNA content of HeLa cells treated with the identified PLK1 inhibitors, DH281, DH285, and DH287. HeLa cells were first incubated in the absence and presence of these compounds at different concentrations (0, 0.12, 0.48 and 1.92 µM) for 48 hr. In the absence of these compounds, only 3.8% of cells exhibited 4N content of DNA. In the presence of 1.92 µM beta-carboline compounds, however, the proportion of HeLa cells in G_2_/M phase reached to 26.7% for DH281, 18.9% for DH285, and 21.0% for DH287. Treatment with a lower concentration of the compounds (0.12 and 0.48 µM) also led to an obvious increase of the cell population in G_2_/M phase (Figure 4A, Table 3). Moreover, the proportion of HeLa cells in G_2_/M phase increased over time after incubation in the presence of 0.8 µM of DH281, DH285 and DH287 (Figure 4B, Table 4). Additionally, the treatment of HeLa cells with these compounds also increased the proportion of cells in S-phase and this increase was dose- and time-dependent (Figure 4A and 4B). We also determined the cell cycle distribution of the non-cancer MRC5 cells after treatment with DH281 at different concentrations. Like HeLa cells, MRC5 showed increased cell population in S-phase after treatment with DH281, but the increase of cells in G_2_/M phase was less significant than HeLa cells (Figure 4C, Table 3), indicating the differential response of non-cancer and cancer cells to the treatment. These data suggest that the treatment of these beta-carboline compounds causes accumulation of cells in S and G_2_/M phases, which is in line with the notion that impaired function of PLK1 leads to compromised DNA replication and mitosis. It is also possible that inhibition of the kinase activity of the whole PLK family by beta-carboline compounds results in the defect in cell cycle progression.

**Table 3:** Cell cycle distribution after treatment with beta-carboline compounds at different concentrations.

Group	HeLa										MRC5			
	Control	DH281			DH285			DH287			Control	DH281		
Dose (µM)	0	0.12	0.48	1.92	0.12	0.48	1.92	0.12	0.48	1.92	0	0.12	0.48	1.92
**G1**	80.6	70.5	55.3	38.4	70.4	55.0	53.4	77.3	64.8	51.9	80.8	75.3	68.2	59.3
**S**	15.5	19.4	30.3	34.9	18.4	28.4	27.7	15.9	21.0	34.7	7.78	9.18	17.3.	25.6
**G2**	3.8	10.1	14.4	26.7	7.61	16.5	18.9	6.86	14.2	21.0	11.4	15.5	14.6	15.1

**Table 4:** Cell cycle distribution of HeLa cells after treatment with DH281, DH285 and DH287 over time.

	Control				DH281				DH285				DH287			
Time (hr)	24	36	48	60	24	36	48	60	24	36	48	60	24	36	48	60
**G1**	70.7	67.9	70.2	68.1	64.1	53.4	42.4	33.9	63.7	56.7	47.3	37.4	64.5	54.6	49.3	30.6
**S**	20.9	20.7	18.1	21.1	25.7	27.7	41.1	51.6	26.9	28.7	36.2	42.5	25.3	29.5	37.8	52.8
**G2**	8.40	11.4	11.7	10.8	10.2	18.9	16.6	14.6	9.44	14.5	16.5	20.2	10.2	15.9	13.0	16.6

**Figure 5:**
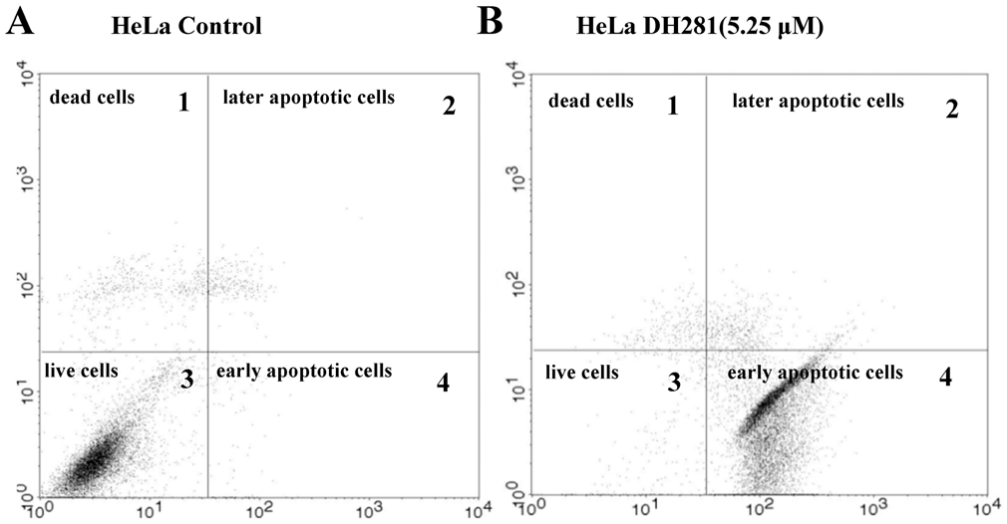
The treatment of HeLa cells with DH281 induces apoptosis. A . The fluorescence profile of untreated HeLa cells. Untreated HeLa cells were collected and incubated with FITC-labeled Annexin V for 10 min, PI for 5 min before flow cytometry analysis. The four quadrants in this figure represent: 1, dead cells (PI positive, FITC negative), 2, late apoptotic cells (PI positive, FITC positive), 3, live cells (PI negative, FITC negative), and 4, early apoptotic cells (PI negative, FITC positive). The apoptotic cells were the combination of early apoptotic and late apoptotic cells (PI^+/−^ FITC^+^). **B**. The fluorescence profile of HeLa cells treated with DH281**.** HeLa cells were exposed to 5.25 µM DH281 for 48 hr. Then the cells were collected and incubated with FITC-labeled Annexin V for 10 min, PI for 5 min before flow cytometry analysis. The four quadrants in this figure are the same as described in panel A.

Previous data suggest that depletion of PLK1 induces apoptosis in cancer cells [32]. Staining with Annexin V–FITC and propidium iodide (PI) is commonly used to distinguish apoptotic, necrotic, and dead cells. PI is impermeant to live cells and apoptotic cells at early stages, but it stains dead cells and apoptotic cells at late stages through its binding to the nucleic acids. Apoptotic cells in early or late stages are stained positively for Annexin V-FITC that binds to phosphotidylserine. We used FACS analysis to determine the proportion of HeLa cells positive for Annexin V-FITC (FITC), PI, or both. The HeLa cells with PI^−/^PI^+^ FITC^+^ were counted as apoptotic cells (Figure 5). Our data indicate that DH281, DH285 and DH287 could induce apoptosis in a dose- and time-dependent manner. For example, only 2.33% (1.62+0.71) of untreated HeLa cells exhibited positive FITC staining, indicating less apoptotic cells. However, 16.19% (3.96+12.23) of HeLa cells became FITC positive after treatment with 1.05 µM DH281 for 48 hr. The treatment with 5.25 µM DH281 induced apoptosis among 89.6% (89.51+0.09) of HeLa cells (Table 5). Moreover, the proportion of FITC positive cells increased over time in the presence of DH281 at 1.21 µM (Table 6). The treatment with DH285 and DH287 also induced apoptosis in HeLa cells in a dose- and time-dependent manner (Table 5 and 6). In contrast to HeLa cells, the induction of apoptosis by DH281 was much less efficient in non-cancer MRC5 cells (Table 5). Therefore, we conclude that the treatment with compounds DH281, DH285 and DH287 induces substantial apoptosis in HeLa cells, but non-cancer MRC5 cells are much less sensitive to the treatment. The distinct apoptotic responses of HeLa and MRC5 cells to beta-carboline compounds could explain the selectivity of PLK1 inhibitors toward cancer cells.

**Table 5:** DH281, DH285 and DH287 induce apoptosis at different concentrations.

Group	HeLa										MRC5			
	Control	DH281			DH285			DH287			Control	DH281		
Dose (µM)	0	0.21	1.05	5.25	0.21	1.05	5.25	0.21	1.05	5.25	0	0.21	1.05	5.25
**PI^+^ FITC^−^**	4.13	3.24	10.22	0.37	2.31	6.03	4.56	3.48	9.81	8.71	0.70	1.63	0.01	10.5
**PI^+^ FITC^+^**	1.62	3.22	3.96	0.09	2.06	0.78	1.19	2.16	5.20	5.58	2.85	5.07	0.59	7.76
**PI^−^ FITC^−^**	93.54	92.57	73.59	10.03	93.91	69.75	38.27	93.25	77.8	20.35	95.9	92.4	86.1	63.2
**PI^−^ FITC^+^**	0.71	0.97	12.23	89.51	1.72	23.44	55.98	1.12	7.20	65.36	1.32	8.95	13.3	18.5

DH281, DH285 and DH287 induce apoptosis at different concentrations. HeLa or MRC5 cells were incubated in the presence of a compound at 0, 0.21, 1.05, and 5.25 µM for 48 hr. After harvest, the cells were stained with FITC-conjugated Annexin V and PI and then subjected to FACS analysis. PI^+^FITC**^−^** represents dead cells; FITC^+^PI^+^ represents late apoptotic cells; FITC**^−^**PI**^−^** represents live cells; PI**^−^**FITC^+^ represents early apoptotic cells. The apoptotic cells are the combination of cells with PI^+^FITC^+^ and PI**^−^**FITC^+^.

**Table 6:** DH281, DH285 and DH287 induce apoptosis over time.

HeLa	Control			DH281			DH285			DH287		
Time (hr)	24	48	72	24	48	72	24	48	72	24	48	72
**PI^+^ FITC^−^**	3.47	3.73	4.11	2.52	3.31	1.41	4.99	6.39	6.03	3.56	7.05	4.56
**PI^+^ FITC^+^**	2.16	2.78	3.88	10.65	8.91	5.97	8.29	10.52	9.73	10.88	8.31	1.19
**PI^−^ FITC^−^**	93.25	92.27	90.36	85.41	71.58	53.08	83.01	70.65	49.75	75.17	58.94	38.27
**PI^−^ FITC^+^**	1.12	1.22	1.65	1.42	16.20	39.54	3.71	12.44	33.49	10.39	25.70	55.98

HeLa cells were incubated in the presence of 1.21 µM of DH281, DH285 and DH287. The cells were harvested at 24, 48 and 72 hr and prepared for FACS analysis. PI^+^FITC**^−^** represents dead cells; FITC^+^PI^+^ represents late apoptotic cells; FITC**^−^**PI**^−^** represents live cells; PI**^−^**FITC^+^ represents early apoptotic cells. The apoptotic cells are the combination of cells with PI^+^FITC^+^ and PI**^−^**FITC^+^.

### DH281, DH285 and DH287 Induce the Formation of Monopolar Spindle in HeLa Cells

**Figure 6:**
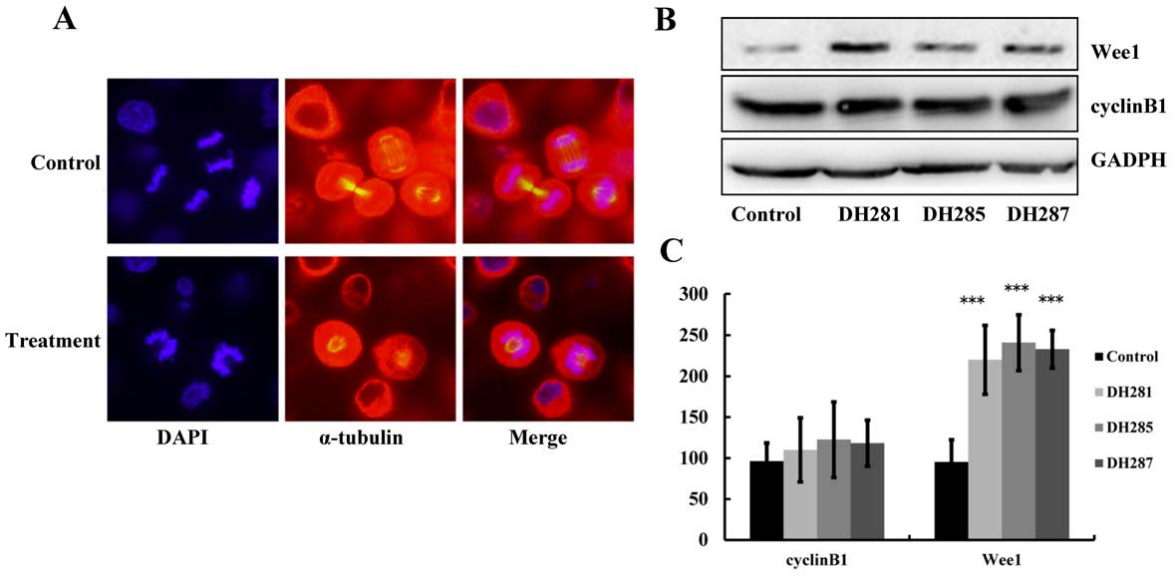
DH281, DH285 and DH287 induce spindle abnormalities and increased Wee1 protein levels in HeLa cells. **A**. HeLa cells were incubated with DH281, DH285 and DH287 at 1.21 µM for 24 hr. After that, the cells were fixed and incubated with FITC-conjugated anti-α-tubulin antibodies (red). DAPI was used to stain nuclear DNA (blue). Cells were visualized and photographed using a confocal microscope with a 20× objective. **B**. The protein levels of Wee1 but not cyclin B1 increase after treatment with the beta-carboline derivatives. HeLa cells were treated with DMSO or DH281, DH285 and DH287 at 1.21 µM for 24 hr and then protein samples were prepared. The proteins levels of Wee1 and cyclin B1 were detected after Western blotting. GAPH is shown as a loading control. **C**. The quantitative analysis of Wee1 and cyclin B levels in HeLa cells treated with β-carboline compounds. HeLa cells were treated with the compounds as described in B and then incubated with anti-Wee1 or anti-cyclin B1 primary antibody and FITC-conjugated secondary antibody. FACS was then performed to measure the protein levels of Wee1 and cyclin B1. Here shows the average of relative protein levels from three separate experiments.

PLK1 plays a pivotal role in centrosome maturation and spindle formation during mitosis [2]. Thus, the down-regulation of PLK1 activity would prevent centrosome maturation as well as the formation of a bipolar spindle. Anti-α-tubulin antibody was used for immunofluorescence staining in order to examine the spindle structure in cells treated with the beta-carboline compounds. After 24 hr treatment with 1.21 µM DH285, many HeLa cells displayed an aberrant monopolar spindle, which is circled by a ring of chromosomes (Figure 6A). DH281 and DH287 also induced formation of monopolar spindles in HeLa cells (data not shown), and this finding is consistent with the observation in cancer cells treated with a known PLK1 inhibitor BI2536 or with PLK1 RNAi [15], [34].

### Cells Treated with DH281, DH285 and DH287 Show Increased Wee1 Protein Level

As the inhibitory kinase for cyclin-dependent kinase (CDK), Wee1 is one of the substrates of PLK1 and this phosphorylation stimulates Wee1 degradation [35]. We expect that the inhibition of PLK1 impairs the phosphorylation of Wee1, thereby resulting in elevated Wee1 protein levels. To test this possibility, we examined the protein levels of Wee1 in HeLa cells after treatment with 1.21 µM beta-carboline compounds for 24 hr. It was obvious that cells treated with DH281, DH285 and DH287 exhibited high levels of Wee1 protein compared to the control cells (Figure 6B). PLK1 also phosphorylates cyclin B1 and this phosphorylation promotes the nuclear localization of cyclin B1 [36]. After treatment with these beta-carboline compounds, a slight increase of cyclin B1 protein was observed (Figure 6B), and this increase could be a consequence of accumulation of cells in G_2_/M phase. However, the change of cyclin B1 levels is much less significant compared to the increase of Wee1 levels after treatment with these compounds. Quantitative analysis of Wee1 and cyclin B protein levels showed the same result (Figure 6C). Because previous work shows that down-regulation of PLK1 causes accumulation of Wee1 protein [23], this result suggests that compounds DH281, DH285 and DH287 inhibit the kinase activity of PLK1 *in vivo*.

## Discussion

PLK1 is an attractive target for cancer treatment because it plays a pivotal role in multiple steps during the cell cycle. It is well documented that PLK1 phosphorylates a subset of proteins to promote centrosome maturation, chromosome bipolar attachment, anaphase entry and cytokinesis [37]. Recent data demonstrate that PLK1 also phosphorylates Orc2 to promote DNA synthesis under stressful growth conditions [33]. Moreover, upregulated PLK1 expression is often observed in tumor cells and this has been shown to be correlated with poor prognosis due to enhanced mitotic activity. Beta-carboline derivatives show significant antitumor activity [38], and we previously demonstrated that a beta-carboline derivative DH166 inhibits the kinase activity of PLK1 in vitro, suggesting that this group of compounds may inhibit the growth of cancer cells by targeting PLK1 [32]. Here, we identified three new beta-carboline derivatives, DH281, DH285 and DH287. Our in vitro kinase assay data indicate that they inhibit the kinase activity of PLK1, as well as that of PLK2 and PLK3.Various researches on the PLK2 transcript and protein levels in different cell types emerge a rather complex pattern, but previous work shows that PLK3 expression decreases during tumor development [23]. Although the beta-carboline compounds can inhibit the kinase activity of PLK1, PLK2 and PLK3, it is likely that the inhibition of PLK1 contributes to toxicity of these compounds to cancer cells [39].

Previous data suggest that beta-carboline derivatives might inhibit the growth of cancer cells through multiple targets such as DNA topoisomerase [40], [41] and cyclin-dependent kinase [42], [43], but our data indicate that PLK1 is likely the key target based on the following observations. First, the strong antitumor activity of DH281, DH285 and DH287 is correlated well with their low IC_50_s for their *in vitro* anti-PLK1 activity. Interestingly, these three compounds also exhibit the highest docking scores, indicating their stronger affinity with the kinase domain of PLK1. Moreover, cells treated with these compounds show obviously elevated protein levels of Wee1, whose phosphorylation by PLK1 is essential for degradation. Importantly, the treatment of HeLa cells with these compounds results in the formation of monopolar spindles, a characteristic of the consequence of PLK1 inhibition [44].

As a rapid developed method, virtual docking was used here to evaluate the interaction between beta-carboline compounds and the PLK1 kinase domain. Interestingly, the high docking scores of DH281, DH285 and DH287 are consistent with their anti-PLK1 activity. Therefore, docking scores could be a very useful tool in evaluating the binding affinity between compounds and target proteins. Based on the docking scores, we could infer that beta-carboline and p-fluro benzyloxy group at position 7 are critical for the anti-PLK1 activity. Also the length and dimensional size of position 9 at beta-carboline could affect the activity to a certain extent.

We previously showed that cancer cells treated with DH166 exhibited elevated cyclin B1 protein levels, but the increase of cyclin B1 was obvious only when cells were treated with high concentrations of DH166 (about 10 fold of IC_50_). Here we found that HeLa cells treated with 1.21 µM DH281, DH285 and DH287 did not show a significant increase of cyclin B1 protein. These cells, however, exhibited a dramatic increase of Wee1 protein levels. We reason that the inhibition of PLK1 by beta-carboline compounds blocks PLK1-dependent Wee1 phosphorylation as well as its degradation. In contrast, the phosphorylation of cyclin B1 by PLK1 does not directly contribute to its degradation but rather regulates its subcellular localization [36]. Because the treatment with beta-carboline derivatives leads to accumulation of cancer cells at G_2_/M stage, wherein the degradation of cyclin B1 is prohibited, the cell cycle arrest induced by high concentration of DH166 may cause elevated cyclin B1. Because the S-phase arrest is more pronounced than G_2_/M arrest after treatment with beta-carboline compounds, the examination of Wee1 protein levels could be a reliable approach to determine the inhibition of PLK1 activity *in vivo*.

It was recently reported that PLK1 knockdown using RNAi only results in a strong mitotic arrest in cancer cells and not in non-cancer cell lines [10]. This notion is supported by our observation that the beta-carboline compounds induced G_2_/M arrest in HeLa cells more efficiently compared to the non-cancer MRC5 cells. In addition, the induction of apoptosis by these compounds in MRC5 cells is less dramatic compared to HeLa cells. It is likely that PLK1 is essential for cell division in cancer cells and the inhibition of PLK1 leads to a more dramatic cell cycle defect, resulting in more apoptotic cells. The difference makes PLK1 an ideal anti-cancer target, because PLK1 inhibitors may selectively kill cancer cells [22].

BI 2536 is a well characterized PLK1 inhibitor. Like BI2536 [44], [45], the identified beta-carboline compounds show strong specificity toward PLKs. Based on previous work, the inhibitory activity of BI 2536 to PLK1 is 4.2- and 10.8-fold stronger than that to PLK2 and PLK3, respectively. In comparison, the IC_50_s of DH281 for PLK1, PLK2 and PLK3 are 0.85, 3.82, and 1.11 µM, respectively. Therefore, the beta-carboline compounds are likely inhibitors for all kinases in the PLK family without obvious selectivity. In contrast to the accumulation of HeLa cells in G_2_/M phase after treatment with BI2536 [45], we observed a clear accumulation of HeLa cells in G_2_/M and S-phases. Although the *in vitro* and *in vivo* evidence indicates the inhibition of PLK1 by both BI2536 and beta-carboline compounds, the later may also target other proteins to induce S-phase arrest. Alternatively, the phosphorylation of some PLK substrates responsible for S-phase progression is more sensitive to beta-carboline compounds. Further investigations are needed to clarify the mechanism that contributes to these differences. In conclusion, three beta-carboline compounds, DH281, DH285 and DH287, were identified with superior anti-PLK1, PLK2 and PLK3 activity. Interestingly, all these compounds inhibit the growth of cancer cells at micromolar concentrations and our future interest is to evaluate their potential as antitumor drugs. We will also perform structure modification in order to enhance the biological activity of these beta-carboline derivatives.

## Materials and Methods

### Reagents and Cell Culture

All the beta-carboline derivatives were provided by Xinjiang Huashidan Pharmaceutical Co. Ltd. The culture media and fetal bovine serum (FBS) were purchased from Hyclone (Hyclone Laboratories Inc., USA). BI2536 was purchased from Selleck (Selleck, USA). All the cell lines were obtained from the Cell Culture Centre, Institute of Basic Medical Science, Chinese Academy of Medical Science and incubated at 37°C supplied with 5% CO_2_. HeLa cells were grown in standard DMEM with 10% FBS. MG63 cells (Human osteosarcoma), HepG2 cells (Human hepatocellular liver carcinoma cell), A549 cells (Human non-small cell lung cancer cell), PC3 cells (Human prostate cancer cell), and MRC5 (Human fetal lung fibroblast cell) were cultured in MEM, MEM, RPMI 1640, Ham’s F-12K, and MEM/NEAA, respectively, supplied with 10% FBS.

### Determination of the Minimum Inhibitory Concentration (MIC) of the Beta-carboline Derivatives using Budding Yeast

The temperature sensitive *cdc5-2* mutants and wild-type strains were used. Saturated yeast cultures were 1/100 diluted into YPD (1% yeast extract, 2% peptone, and 2% glucose) medium containing beta-carboline compounds at different concentration: 40, 20, 10, 5, 2.5, 1.25, 0.625, and 0 µg/ml. After incubation at 25°C for 24 hr, the growth of the yeast cells was determined by measuring OD_600_.

### Cell Proliferation Assay

MRC5, HeLa, HepG2, PC3 and MG63 cells were seeded at 3000–6000 cells/well (200 µl/well) in a 96-well plate and incubated overnight. Then different concentrations of compounds were added into the cell cultures with a final concentration of 0.1% DMSO and incubated for an additional 48 hr. After that, 22 µl MTT solution (5 mg/ml) was added into each well and incubated at 37°C for 4 hr. The supernatant was removed, 150 µl DMSO was added into each well to dissolve the MTT formazan, and the plates were then examined with PE Envison 2104 at 560 nm. The IC_50_ was defined as the concentration that induces 50% cellular death in comparison with untreated controls and calculated by Origin pro7.5 program based on the following formula: (Absorbance_control_ – Absorbance_test_)/Absorbance_control_ × 100.

### *In vitro* Kinase Assay for PLK1, PLK2, PLK3, Aurora A, Aurora B, CDK1/CyclinB(Human) and CDK2/CyclinE(Human)

The inhibition of PLK1 kinase activity was assessed using the Cyclex PLK1 Assay/Inhibitor Screening Kit (MBL International Corporation, Japan). The kit includes anti-phospho-threonine polyclonal antibody (PPT-07) and peroxidase coupled anti-rabbit IgG antibody as a reporter in a 96-well ELSIA format. Compounds were used at the concentrations from 10**^−^** ^3^ to 10^2^ µg/ml and the absorbance was determined at 450 nm. A similar protocol was used to determine the kinase inhibition for PLK2, PLK3, Aurora A and Aurora B. The kinase activity of CDK1/cyclinB and CDK2/cyclinE was analyzed in the presence of serially diluted inhibitors. For each reaction, 20 ng of recombinant kinase was used and 10 mg casein from bovine milk (Sigma) was added as the kinase substrate. The kinase reaction buffer contains 8 mM MOPS pH7.0, 0.2 mM EDTA, 0.1 mg/ml histone H1, 10 mM and MgAcetace and [γ**^−^** ^33^P-ATP] (500 cpm/pmol). The reaction was initiated by adding the MgATP mix. After incubation for 40 minutes at room temperature, the reaction was stopped by the addition of 3% phosphoric acid solution. We spotted 10 µl of the reaction mixture onto a P30 filtermat and washed three times for 5 min with 75 mM phosphoric acid and once with methanol prior to drying and scintillation counting.

### Molecular Docking

To evaluate the activity of the selected compounds, the docking program FlexX (SYBYL7.3, Tripos Inc.) was used to dock PLK1 (PDB code: 2rku). First, all crystal water molecules were removed from the original structure, hydrogen was added using Biopolymer module in SYBYL, and standard AMBER atomic partial charges were assigned. To obtain an optimal starting conformation, all ligands were minimized using Tripos standard force field and Gasteiger-Hückel atomic partial charges with termination gradient to reach the lowest energy state before docking. During the docking, the ATP-binding pocket was the focus of our interest and the active sites were defined as all the atoms within a radius of 6.5 Å in the pocket. During soft docking simulations, the free energy of binding was calculated mainly by the sum of hydrogen bonds and hydrophobic interactions. The sum of the lowest estimated free energy from various binding conformations of each ligand was calculated and ranked by CScore function in SYBYL with default variables.

### Fluorescence-activated Cell Sorter (FACS) Analysis

Tumor cells were exposed to vehicle (0.1% DMSO) or compounds at 0, 0.12, 0.48, and 1.92 µM for 48 hr or 0.8 µM for 24, 48, and 72 hr; MRC5 cells were exposed to compounds at 0, 0.12, 0.48, and 1.92 µM for 48 hr. The floated and attached cells were treated by trypsinization and washed with cold PBS. The cells were then fixed with 70% (v/v) ethanol at 4°C overnight. The fixed cells were washed twice with PBS and incubated with 50 µg/mL RNase A (Sigma, USA) and 50 µg/mL propidium iodide (PI) (Sigma, USA) at 37°C for 30 min.

### FITC-Annexin V/PI Apoptosis Assay

HeLa cells were either treated with 0, 0.21, 1.05 and 5.25 µM of DH281, DH285 and DH287 for 48 hr, or treated with 1.21 µM compounds for 24, 48 and 72 hr, while MRC5 cells were exposed to compounds at 0, 0.21, 1.05, and 5.25 µM for 48 hr. The cultured cells were harvested, washed three times with PBS, and resuspended in 500 µl binding buffer (10 mM Hepes/sodium hydroxide (pH7.4), 140 mM sodium chloride, and 2.5 mM CaCl_2_). Then 5 µl FITC-labeled Annexin V (Zhongshan JinQiao Biotechnology Ltd., Beijing) was added and the cells were incubated in the dark for 10 min. We added 5 µl of propidium iodide (PI) (10 µg/ml in binding buffer) to each sample before flow cytometry analysis.

### Immunofluorescence Microscopy

HeLa cells were collected and then grown onto cover-slips. After 3 day treatment with compounds, cells were rinsed twice with PBS and then fixed by immersing the cover-slips in the immune-staining fixing solution (Beyotime, P0098) overnight. After washed with washing buffer (Beyotime, P0106) for 3 times, the cells were incubated with blocking buffer (Beyotime, P0102) for 60 min. The primary antibody anti-α-tubulin (Abcam) was used at 1∶200 dilutions; H&L (Cy5)-labeled secondary antibody was used at 1∶200 dilutions. The cover-slips were then mounted with PBS:glycerol (1∶1) mounting medium containing DAPI and examined under an inverted confocal microscope. Images were analyzed by the 3D image software Volocity (Improvision).

### Western-Blotting Analysis

HeLa cells were incubated with or without 1.21 µM DH281, DH285 and DH287 for 24 hr. The cells were washed twice with cold 1×PBS and lysed in RAPI buffer (Applygen technologies Inc.) for 30 min and centrifuged at 14,000 rpm for 20 min at 4°C. We separated 15 µl of total protein (1 µg/ µl) with 12% SDS-PAGE gels and transferred the proteins to PVDF membrane. After blocking with 5% (W/V) nonfat dry milk in Tris-buffered saline containing 0.2% Tween-20 (TBST) for 1 hr, the membrane was incubated with desired primary antibodies for 2 hr and then with peroxidase-conjugated secondary antibodies for 1 hr. The protein bands were detected using ECL System (Piscataway, New Jersey, USA). The level of GADPH was used as a loading control. The antibodies against Wee1 were purchased from Cell Signaling Technology, Inc.; and the antibodies against cyclin B1, PLK1 and GADPH were purchased from Abcam.
